# Incidence of Intracranial Hemorrhage After a Cranial Operation

**DOI:** 10.7759/cureus.616

**Published:** 2016-05-20

**Authors:** Virendra R Desai, Robert Grossman, Harlan Sparrow

**Affiliations:** 1 Neurosurgery, Houston Methodist Neurological Institute; 2 Neurosurgery, Houston Methodist

**Keywords:** hematoma, hemorrhage, intracranial, post-operative

## Abstract

**Objective:**

To describe the characteristics of patients who underwent a cranial operation and postoperatively suffered an intracranial hemorrhage significant enough to require evacuation.

**Materials & methods:**

3,109 cranial operations were performed at Houston Methodist Hospital (Texas Medical Center campus) between January 2009 and December 2013. Of these, 59 cases required a second operation for evacuation of an intracranial hemorrhage. The information gathered included the patients’ age, gender, past medical history, medications and laboratory data, initial diagnosis, date/type of first and second operations, duration of hospitalization, discharge condition, and discharge destination.

**Results:**

The study found a 1.90% rate of a postoperative hemorrhage significant enough to require evacuation after a cranial operation. The average age in the cohort requiring reoperation was 63 +/- 14 years with 42 male and 17 female. Hematoma evacuations were performed at various time intervals depending on the pathology treated at the initial operation. The time to second operation was 2.7 days after intraparenchymal hematoma evacuation, 6.0 days after cerebrovascular surgery, 6.2 days after tumor surgery and 9.7 days after subdural hematoma evacuation. The rate of postoperative hematoma development was 9.1% after a subdural hematoma evacuation, while it was only 1.1% in all other operations. Overall, those requiring hematoma evacuation had a 15% mortality rate, 64% were non-ambulatory, and 54% were discharged to long-term acute care facility, skilled nursing facility, rehabilitation facility or hospice.

**Conclusions:**

Neurological outcomes were poor in patients who underwent a cranial operation and required a second operation to remove a hematoma. This study suggests close observation of elderly males after a cranial operation, especially after subdural hematoma evacuation, and longer observation time for patients undergoing subdural hematoma evacuation than intraparenchymal hematoma evacuation, tumor surgery or cerebrovascular surgery.

## Introduction

Intracranial hemorrhage (ICH) after a cranial operation is an uncommon but potentially serious adverse event. The ability to preoperatively determine which patients are at high risk of ICH and the ability to quickly diagnose and treat ICH is of paramount importance, since the quicker the hemorrhage can be evacuated, the greater the likelihood of functional recovery. Toward this goal the objective of this study was to delineate which patients suffer hemorrhages after a cranial operation, what medical comorbidities such patients suffer, when the hemorrhage required evacuation, what methods were used to treat the hemorrhage, and what the neurological outcomes of the patients were. Informed consent was obtained from all patients undergoing an operation at Houston Methodist Hospital, and IRB approval was obtained for this study.

## Materials and methods

A retrospective analysis was performed of patients who had a cranial operation at Houston Methodist Hospital (Texas Medical Center campus) from January 2009 to December 2013 and underwent reoperation for evacuation of a hematoma within 30 days of their initial operation. This list of patients was compiled via coding analysis. Surgeries included supratentorial craniotomy, suboccipital craniotomy, burr hole(s), ventriculoperitoneal shunt placement, deep brain stimulation, and trans-sphenoidal surgery. Only those patients who required a second operation within 30 days to evacuate a hematoma were included in this study. In this time period, 3,109 cranial operations were performed, with 59 cases suffering postoperative hemorrhage requiring evacuation. The following information was gathered via individual chart review:

Type of hemorrhage including epidural, subdural, intraparenchymal, and mixed.

Patient characteristics including age; gender; past medical history including hypertension, diabetes mellitus, coronary artery disease, coagulopathy, end-stage renal disease on hemodialysis, and liver failure—defined as liver disease advanced enough to cause INR >1.5 and/or platelet count <100,000.

Anticoagulant or antiplatelet use and type, the admission and preoperative coagulation laboratory values and platelet counts.

Details of the diagnosis and operations including initial diagnosis; the date and purpose of the first operation; the urgency of the first operation—elective versus emergent; the location of the intracranial hemorrhage; and the date and type of second operation.

Long-term outcome of each patient including duration of hospitalization, combining the duration of the initial and subsequent hospitalization if the patient was discharged in-between the two operations; morbidity requiring tracheostomy and/or percutaneous endoscopic gastrostomy tube placement; ambulatory status; discharge destination; and death.

Of note, none of the patients had an intrinsic coagulopathy.

## Results

A total of 59 (1.9%) cases out of 3,109 cranial operations between January 2009 and December 2013 were identified as requiring a second operation to evacuate an ICH within 30 days of the initial operation.

Table 1 represents the characteristics such as average age, medical problems, and medication use of the patients undergoing reoperation for evacuation of a postoperative hematoma.

**Table 1:** Demographic Data

Age +/- SD	62.75 +/- 14.02
Gender Breakdown (M:F)	42:17
Hypertension	44 (75%)
Diabetes Mellitus	25 (42%)
Coronary Artery Disease	13 (22%)
End-stage Renal Disease on Hemodialysis	7 (12%)
Liver Failure	4 (7%)
Anticoagulant Use	12 (20%)
Antiplatelet Use	21 (36%)

Table 2 shows the laboratory values such as partial thromboplastin time, prothrombin time, INR, and platelet count of those requiring reoperation for hematoma evacuation.

**Table 2:** Laboratory Values (mean +/- SD)

	Partial Thromboplastin Time (Seconds)	Prothrombin Time (Seconds)	INR	Platelet Count (Thousand per Microliter)
Admission	33.0 +/- 11.6	15.9 +/- 5.1	1.3 +/- 0.5	220 +/- 98
Immediately preoperative	29.7 +/- 4.7	14.1 +/- 0.9	1.1 +/- 0.1	209 +/- 79

Tables 3-5 depict the initial operation characteristics, including preoperative diagnosis, type of operation, and urgency. The "other" category included two hemicraniectomies, one decompression for Chiari malformation, one biopsy of an intracranial lesion, one cranioplasty, and one wound debridement and revision.

**Table 3:** Initial Operation Diagnosis Types

Diagnosis	Number of Cases	Percent of Cases
Subdural Hematoma	27	46%
Tumor	11	19%
Vascular	8	14%
Other	6	10%
Intraparenchymal Hematoma	5	8%
Hydrocephalus	1	2%
Functional	1	2%

**Table 4:** Initial Operation Procedure Types

Procedure	Number of Cases	Percent of Cases
Supratentorial Craniotomy	35	59%
Burr Holes	15	25%
Suboccipital Craniotomy	5	9%
Trans-sphenoidal	2	3%
Ventriculoperitoneal Shunt	1	2%
Deep Brain Stimulation	1	2%

**Table 5:** Initial Operation Scheduling

	Number of Cases	Percent of Cases
Elective	43	73%
Emergent	16	27%

Table 6 presents tumor sub-categories, intra-axial versus extra-axial.

**Table 6:** Tumor Location

	Number of Cases	Percent of Cases
Intra-axial	6	55%
Extra-axial	5	45%

Tables 7-9 show characteristics of the second craniotomy, including location of hematoma and average time between initial and second operations. The average time between the initial and second operations was related to the pathology for which the surgery was performed. If the second craniotomy was performed on the same day as the initial operation, the average time was designated as 0.5 days. Hydrocephalus and functional cases are not included in these tables because there was only one case of each requiring hematoma evacuation. The locations of postoperative hematomas were related to the initial diagnosis. For patients who underwent an initial operation for subdural hematoma evacuation, 96% of postoperative hematomas were recurrent subdural hematomas and four percent were located in other compartments.

**Table 7:** Second Operation Hematoma Location

Hematoma Location	Number of Cases	Percent of Cases
Subdural	30	51%
Intraparenchymal	13	22%
Epidural	7	12%
Multiple	6	10%
Sella	2	3%
Intraventricular	1	2%

**Table 8:** Time to Second Operation based on Initial Diagnosis

Initial Diagnosis	Time to Second Operation (Days)	Time to Second Operation (Interquartile Range)
Subdural hematoma	9.7	3 - 16
Tumor	6.4	1 - 15
Vascular	6.0	1.25 - 9
Other	12.6	4.63 - 20
Intraparenchymal	2.7	0.75 - 5
Hydrocephalus	4.0	-
Functional	23.0	-

**Table 9:** Second Operation Hematoma Location based on Initial Diagnosis

Initial Diagnosis	Epidural	Subdural	Intraparenchymal	Intraventricular	Sella	Multiple
Subdural hematoma	-	26 (96%)	-	-	-	1 (4%)
Tumor	3 (25%)	-	5 (42%)	-	2 (17%)	1 (8%)
Vascular	-	1 (13%)	5 (63%)	-	-	2 (25%)
Other	2 (33%)	2 (33%)	1 (17%)	-	-	1 (17%)
IPH	2 (40%)	-	2 (40%)	-	-	1 (20%)
HCP	-	-	-	1 (100%)	-	-
Functional	-	1 (100%)	-	-	-	-

Table 10 shows the 30-day outcomes and mortality rate.

**Table 10:** Long-term Outcome

	Number of Cases	Percent of Cases
Non-ambulatory	38	64%
PEG	20	34%
Tracheostomy	17	29%
Mortality	9	15%

Table 11 shows the duration of hospitalization.

**Table 11:** Duration of Hospitalization

	Number of Cases	Percent of Cases
<1 week	6	10%
1-2 weeks	8	14%
2-3 weeks	15	25%
3-4 weeks	10	17%
>4 weeks	20	34%

Table 12 shows the discharge destinations.

**Table 12:** Discharge Destination

	Number of Cases	Percent of Cases
Home	18	31%
Long-Term Acute Care	16	27%
Death	9	15%
Skilled Nursing Facility	7	12%
Rehab	7	12%
Hospice	2	3%

Table 13 depicts the subgroup analysis of rates of postoperative hematoma formation by preoperative diagnosis. 1,207 of the total 3,109 cases (39%) were for tumor resection, and 12 of these cases (1.0%) required a second operation for postoperative hemorrhage evacuation. 439 of the total 3,109 cases (14%) were functional cases, largely deep brain stimulator electrode placement and microvascular decompressions, and only one of these (0.2%) required a second operation for postoperative hemorrhage evacuation. 433 cases (14%) were for cerebrospinal fluid (CSF) diversion, of which one (0.2%) required a postoperative hemorrhage evacuation. 393 cases (13%) were classified in our “other” category, consisting largely of hemicraniectomies, seizure/epilepsy cases, traumatic skull fractures, CSF leaks, wound infections, and suboccipital craniectomies for chiari malformation. Six of these cases in the other category (1.5%) required a postoperative hemorrhage evacuation. 296 cases (9.5%) were performed initially for subdural hematoma evacuation, and 27 of these (9.1%) required a postoperative hemorrhage evacuation. 278 cases (8.9%) were vascular cases, of which eight (2.9%) required a postoperative hemorrhage evacuation. 63 cases (2.0%) were for intraparenchymal hematoma evacuation, of which five (7.9%) required a postoperative hemorrhage evacuation.

**Table 13:** Hematoma Evacuation Rates by Preoperative Diagnosis

Preoperative Diagnosis	Percentage of Total Craniotomies (%)	Percentage Requiring Reoperation for Hematoma Evacuation (%)
Tumor	38.82	0.99
Functional	14.12	0.23
Hydrocephalus	13.93	0.23
Other	12.64	1.53
Subdural hematoma	9.52	9.12
Vascular	8.94	2.88
Intraparenchymal hematoma	2.03	7.94

## Discussion

### Rates of hematoma development

Rates of postoperative hemorrhage after intracranial surgery vary greatly in the literature, ranging from 0.77–50% [1-4], but the definition of postoperative hemorrhage varies as well, making comparisons between studies difficult. Some studies defined it as any hemorrhage within the operative bed. However, some residual hemorrhage can be expected and be clinically benign. Other studies defined it as hemorrhage significant enough to require surgical evacuation, as we have defined it in our study [1]. Studies that examined patients who clinically deteriorated reported rates of 0.77-6.9% with postoperative hematoma; those that included patients with any radiographic evidence of hemorrhage reported rates of 10.8-50% [1-3]. The present study found a 1.9% rate of postoperative hematoma development requiring evacuation, tending towards the lower end of the incidence of postoperative hematomas reported in the literature. Considering recurrent subdural hematomas as a separate category, we found a 9.1% rate of postoperative hematoma development after subdural hematoma evacuation and a 1.1% combined rate in the remaining categories.

### Location of postoperative hematoma

Many studies have found that the majority of postoperative hematomas were epidural or intraparenchymal, while in the present study, the most common location was subdural [1-3, 5-6]. In the present study, the postoperative hematoma location was highly dependent on the preoperative pathology. While 96% of recurrent hematomas after subdural hematoma evacuation occurred in the subdural space, the remainder of the cranial operations largely resulted in intraparenchymal hematomas.

### Timing of postoperative hematoma development

Gerlach et al. found that the majority of their hematomas were discovered within three days of the initial operation [5]. Kalfas et al. found 35% of their ICH cases were discovered within 12 hours [4]. Taylor et al. found that of 2,305 patients, 50 developed postoperative hematomas with 44 forming within six hours. They conclude that elective supratentorial operations can be observed in the ICU for six hours but shouldn’t need longer observation. This does not apply to emergency cases in which life-threatening, space-occupying lesions have been removed, or in posterior fossa cases that carry the risk of hydrocephalus, cranial nerve palsies and respiratory/airway issues if hemorrhage/swelling were to occur [6].

Of the 59 cases in the present study requiring reoperation for hematoma evacuation, 46% were for recurrent subdural hematomas. When the initial operation was for evacuation of intraparenchymal hematoma, return to the operating room for evacuation of a postoperative hematoma occurred sooner (in 2.7 days) than any other type of operation. “Other” operations, including hemicraniectomy and anterior temporal lobectomy for malignant cerebral edema, multiple biopsies of viral lesions, Chiari malformation, cranioplasty, and scalp wound debridement and revision, had the longest interval between first and second operations at 12.6 days. Recurrent subdural hematomas typically had reoperation after 9.7 days.

The present study found a longer interval between initial operation and hematoma evacuation than in the literature. There are several possible explanations. First, the studies cited above provided the time from initial operation to discovery of the hematoma, whether it was via clinical deterioration of the patient or routine postoperative imaging. In the present study, we report the time between initial operation and subsequent operation, although the hematoma may have been discovered earlier.

### Risk factors for hematoma development

Various risk factors have been analyzed for predisposing to hematoma development: hypertension was generally found to be significant in most studies while diabetes mellitus, cerebral amyloid angiopathy and atherosclerosis were generally not significant [1, 4]. Basali et al. noted that patients with postoperative intracranial hemorrhage had a significantly greater rate of intraoperative and postoperative/prehemorrhage hypertension [3]. Intraoperative hypertension occurs during brain manipulation, head pin application, use of epinephrine-containing anesthetics, periosteal dissection and emergence. Basali et al. also found a significantly high odds ratio for ICH when patients had BP <160/90 intraoperatively but then had elevated BP postoperatively. This suggests some vessels may not have been adequately tested intraoperatively for the possibility of leaking when blood pressure rises [3].

Age is a significant risk factor for hematoma development—six-fold more likely at age over 70 and 12-fold over 75 [5, 7]. Other risk factors include antiplatelet use, including aspirin and non-steroidal anti-inflammatory drugs, preoperative mannitol administration, excessive alcohol use, coagulopathies, disseminated intravascular coagulation, thrombocytopenia, acute decreases in platelet counts postoperatively, excessive intraoperative blood loss, poor response to preoperative platelet transfusions, factor XIII deficiencies, and decreased fibrinogen levels [1-2, 5, 7-11].

### Long-term outcomes of patients undergoing repeat procedure for hematoma evacuation

Outcomes in prior studies have been categorized generally by Glasgow Outcome Scale (GOS) at three months or longer and by mortality rates. Generally, outcome is poor with a range of mortality from 13-41% depending on the type of initial operation (tumor versus traumatic ICH, etc.). Good recovery, defined as GOS 4 or 5, has been reported in the range of 39-71% [2, 4-5, 10].

In the present study, we found a 15% mortality rate while hospitalized. About one third of patients required a tracheostomy and/or percutaneous endoscopic gastrostomy. Nearly two thirds were non-ambulatory and over half were discharged to long-term acute care facility (27%), skilled nursing facility (12%), rehabilitation (12%) or hospice (3%).

While most studies have reported neurological outcomes in terms of GOS and evaluated patients three months after operation, in the present study, we observed only their status at discharge from the hospital which was most often several weeks after hematoma evacuation. Our outcomes being short-term thus appear to be on the worse end of the spectrum. For similar reasons, our mortality rate may appear towards the lower end of the literature rate possibly secondary to evaluation only while hospitalized. 

### Limitations

The study was retrospective, using coding analysis to compile a list of cranial cases who had hematoma evacuation. Some cases may have been missed, since only a limited number of search terms were used—only cases that had “craniotomy” and “hemorrhage” both documented in the chart were initially included. After this, we performed a thorough chart review, and thus no further cases were missed.

The present study reported postoperative outcomes only, rather than comparing this with the patients’ preoperative status. Thus, we cannot precisely quantify the extent to which the patients’ outcomes were worsened secondary to reoperation for hemorrhage evacuation.

Because this was a descriptive study, no statements can be made to compare these patients who had evacuation of a postoperative ICH with those not requiring a repeat cranial procedure. The medical comorbidities and laboratory values noted in this study only describe the patients in this study and do not reflect the characteristics of patients not requiring a repeat operation. Thus, we cannot determine the risk of hematoma occurrence based on a patient’s medical comorbidities and laboratory values, as we have no comparison to those patients not requiring a repeat operation.

## Conclusions

Neurological function is generally poor in patients who undergo a cranial operation and require a second operation to remove a hematoma. This study suggests closer observation of elderly males after a cranial operation, especially after subdural hematoma evacuation, and longer observation time for patients undergoing subdural hematoma evacuation than intraparenchymal hematoma evacuation, tumor surgery or cerebrovascular surgery.
